# Employees Perception of Organizational Crises and Their Reactions to Them – A Norwegian Organizational Case Study

**DOI:** 10.3389/fpsyg.2022.818422

**Published:** 2022-08-10

**Authors:** Jarle Løwe Sørensen, Jamie Ranse, Lesley Gray, Amir Khorram-Manesh, Krzysztof Goniewicz, Attila J. Hertelendy

**Affiliations:** ^1^Department of Business, History and Social Sciences, USN School of Business, University of South-Eastern Norway, Kongsberg, Norway; ^2^School of Nursing and Midwifery, Griffith University, Gold Coast, QLD, Australia; ^3^Department of Emergency Medicine, Gold Coast Health, Gold Coast, QLD, Australia; ^4^Department of Primary Health Care and General Practice, University of Otago, Wellington, New Zealand; ^5^Joint Centre for Disaster Research, Massey University, Wellington, New Zealand; ^6^Department of Surgery, Institute of Clinical Sciences, Sahlgrenska Academy, Gothenburg University, Gothenburg, Sweden; ^7^Gothenburg Emergency Medicine Research Group (GEMREG), Sahlgrenska Academy, Gothenburg University, Gothenburg, Sweden; ^8^Department of Security, Polish Air Force University, Dęblin, Poland; ^9^Department of Emergency Medicine, Fellowship in Disaster Medicine, Beth Israel Deaconess Medical Center and Harvard Medical School, Boston, MA, United States; ^10^Department of Emergency Medicine, Harvard Medical School, Boston, MA, United States; ^11^Department of Information Systems and Business Analytics, College of Business, Florida International University, Miami, FL, United States

**Keywords:** crisis communication, crisis management, organization, organizational psychology, sensemaking

## Abstract

Organizational sensemaking is crucial for resource planning and crisis management since facing complex strategic problems that exceed their capacity and ability, such as crises, forces organizations to engage in inter-organizational collaboration, which leads to obtaining individual and diverse perspectives to comprehend the issues and find solutions. This online qualitative survey study examines how Norwegian Sea Rescue Society employees perceived the concept of an organizational crisis and how they sensed their co-workers react to it. The scope was the ongoing COVID-19 pandemic, a global event affecting all countries and organizations and responding similarly globally. Data were collected during the Fall of 2020. The instrument of choice was the Internal Crisis Management and Crisis Communication survey (ICMCC). The results showed that the overall sample strongly believed in their organization’s overall resilience level. However, a somewhat vague understanding of roles and responsibilities in a crisis where detected, together with some signs of informal communication, rumor spreading, misunderstanding, frustration, and insecurity. This study contributes to the academic field of organizational research, hence crisis management and sensemaking, and could be valuable to managers and decision-makers across sectors. Increased knowledge about how employees react to a crisis may help optimize internal crisis management planning and utilize robust mitigation and response strategies.

## Introduction

On March 12, 2020, the Norwegian Institute of Public Health (2020) reported that 621 individuals had tested positive for the SARS-CoV-2 (COVID-19) coronavirus disease. In response, the Norwegian Government introduced the most substantial and intrusive control measures in Norway since World War 2. Kindergartens, schools, and higher education institutions were closed, cultural, sports events and organized activities were prohibited, and health professionals who work in patient care were forbidden to leave the country. Additionally, entry quarantine to Norway and a ban on staying on one’s leisure property were introduced (Norwegian Government, 2020). One year later, COVID-19 has taken its toll on both societies and individuals. The virus has also brought several unexpected variables for organizations, which many people had not planned for or were equipped to handle. Against this backdrop, we extend Bailey and Breslin's (2021) argument that an organization’s ability to mitigate and respond to major crises like the COVID-19 pandemic is closely linked with the concept of resilience. Here, organizational resilience relates to an organization’s capacity to learn and reflect from past incidents, show flexibility, adapt to new situations, and take advantage of- and utilize existing resources (Steen and Morsut, 2020). Considering that nations have always struggled with and fought against infectious diseases, it could be assumed that the ability to mitigate from- and respond to pandemics has continuously increased, but as Klein (2021) pointed out, more knowledge alone has not always shown enough. Imposed measures must also be explained and put in the proper social perspective. The ongoing pandemic has taught us that a crisis may affect an organization’s external and internal life. Therefore, it is in an organization essential to build and join together both inner and outer resilience in crisis management. A paucity of literature reviews the dynamics of internal crisis management (Frandsen and Johansen, 2011; Heide and Simonsson, 2015; Adamu and Mohamad, 2019).

Organizational sensemaking is crucial for resource planning and crisis management. Previous publications indicate that when organizations face complex strategic problems which exceed their capacity and ability, such as diverse crises, they search for inter-organizational collaboration, which enables the accumulation of staff individual and diverse perspectives, in the hope of understanding the depth and nature of the issues to find proper solutions (Seidl and Werle, 2018; Tan et al., 2020). This online qualitative survey study examines how Norwegian Sea Rescue Society employees perceived the concept of an organizational crisis and how they sensed their co-workers react to it. The scope was the ongoing COVID-19 pandemic, a global event affecting all countries and organizations and responding similarly globally. As a contribution to organizational research, this online-survey-based case study, from a sensemaking perspective, aimed to examine how Norwegian Sea Rescue Society employees perceived the concept of an organizational crisis (the COVID-19 pandemic) and how they sense their co-workers reacted to it. Karl Weick’s theory on sensemaking (Weick, 1995, 2010, 2012, 2015) was deemed most appropriate as it focuses on crises and situations where organizational sensemaking is challenged or breaks down (Johansen et al., 2012).

As the sensemaking concept, today can be described more as a general notion than a unified term; we see the need to narrow down the scope in this study and have chosen Maitlis and Christianson's (2014) four recurring sensemaking themes as the starting point for our discussion. The COVID-19 pandemic was proven to affect all nations and organizations which responded to its progression and impacts somewhat similarly and globally (Khorram-Manesh et al., 2020). We thus consider this study to have relevant transfer value across sectors. Increased knowledge about how individuals make sense of- and react to organizational crises would contribute to organizational research and sensemaking. This online qualitative survey study showed that the overall sample strongly believed in their organization’s overall resilience level. However, a somewhat vague understanding of roles and responsibilities in a crisis where detected, together with some signs of informal communication, rumor spreading, misunderstanding, frustration, and insecurity. Our research is based on the frameworks of an earlier Danish study on internal crisis management and communication. Internal response, also known as an organizational response or business continuity management, focuses on an organization’s inner dynamics to a crisis, hence its overall approach and strategic instruments (Johansen et al., 2012). We consider addressing the internal perspective especially relevant as last decade, CM and CC research have, in large, focused on the external dimensions of the crisis, hence, how to restore from a possible reputation or image damage. Thus, research on the internal life in crisis from a sensemaking perspective is to be considered limited (Frandsen and Johansen, 2011).

## Background

### Crisis

A crisis can be described as an overwhelming situation that overstrains available capacities and resources (Van Wart and Kapucu, 2011; Sriharan et al., 2022). A crisis is more transboundary than everyday emergencies and often exceeds natural or manmade geographical, organizational or administrative borders (Ansell et al., 2010). Despite growing attention to the crisis, it has been proven difficult to establish a consensus about a unified crisis definition (Wolbers et al., 2021). Bundy et al. (2017) point out that research on crises and their management remains fragmented. The organizational crisis literature is somewhat cofounded by several and sometimes conflicting explanations and definitions (Kovoor-Misra et al., 2001), and there are still many theoretical, practical, and conceptual challenges that need resolving (Lalonde and Roux-Dufort, 2013). According to Roux-Dufort and Lalonde (2013), the diversity of conceptualizations indicates that we are faced with a wandering phenomenon. Upon examining the development of- and knowledge gaps in business and management research on organizational resilience, Linnenluecke (2017) found in her review that the research on resilience has developed into five main streams: “(1) organizational responses to external threats, (2) organizational reliability, (3) employee strengths, (4) the adaptability of business models or (5) design principles that reduce supply chain vulnerabilities and disruptions” (p. 4). The review concluded that many organizations, as a result, will face unpreparedness when a crisis strike if they do not increase their overall capacities and their knowledge about the discussion-making process and resilience planning.

One type of crisis, organizational crisis, an area of interest in this study, is defined as a low-probability, high-impact event that threatens the organization’s sustainability (Pearson and Clair, 1998; Kim, 2018). It can be caused by natural and manmade hazards and pose internal and external challenges (Winston, 2019). Other earlier sources, such as Nystrom and Starbuck (2015), viewed crises as a threat to organizational development, while Milburn et al. (1983) saw them both as a threat and an opportunity. While Weick (1988) argued that a crisis was a low probability event, others like Smart and Vertinsky (1984) reasoned that it could be high and low. Whereas Pearson and Mitroff (1993) claimed that the element of surprise was a hallmark in crisis, Kerchner and Schuster (1982), on the contrary, argued that they were somewhat predictable. Relevant to this study, and as pointed out by Boin and t Hart (2022), a crisis could also be seen as a “*catalyst for change*” (p. 13). The justification argument stems from what Barton (1970) referred to as “collective stress,” which, summarized by Boin and t Hart, helps relieve and wash away existing and often tradition-based institutional myths and patterns. New thinking may be beneficial but also challenging for organizations that have a markedly mechanistic (Burns and Stalker, 1961) approach to crisis management, where the action logic is focused on a linear, rigid, and fixed hierarchical system.

From an organizational viewpoint, mitigating and preparing for crises has become increasingly tricky as 21st-century organizations face ever-changing technological, communicative and cultural challenges (Aljuhmani and Emeagwali, 2017). Crisis survival is thus much dependent on the organization’s overall level of resilience (Teo et al., 2017). Resilience is the ability to react (Williams et al., 2017) and recover from damaging events or blows (Longstaff, 2005). Hwang and Lichtenthal (2000) argued that organizations could be subject to two main types of crisis; abrupt or cumulative, where the abrupt is a one-time event that occurs suddenly and challenges the state of normal, while the cumulative builds up gradually over time. Following the latter, an organizational crisis may be viewed as a three-stage multi-disciplinary process consisting of pre-crisis, crisis, and post-crisis (Johansen and Frandsen, 2007). The organization identifies and takes strategical and tactical mitigation measures during this process, responds to the crises, and restores a normal state (Coombs and Holladay, 2014; Zamoum and Gorpe, 2018). While such a multi-disciplinary approach to organizational crisis handling is embraced by several scholars (Smart and Vertinsky, 1984; Weick, 1988; Kovoor-Misra et al., 2001), it has also been criticized for nurturing up under and contributing to lacking unification within the field of organizational crisis research (Pearson and Clair, 1998).

### Managing Crisis

Compared to managing routine-based daily operations, a crisis (also known as a disaster) can be quite challenging and stressful (Peyravi et al., 2021). Stated reasons include that crisis occurs with less regularity and often is more disorganized and resource-overwhelming (Ansell et al., 2010; Sørensen, 2017). It also presents stakeholders with higher uncertainty levels (Mitroff et al., 1987), combined with pressure to make quick and effective decisions within short time frames, often based on little or poorly validated information (Lu and Xue, 2016). Lastly, as Van Wart and Kapucu (2011) pointed out, there is an additional inconsistency and notable difference in how the crisis concept is viewed across public and private sectors. While public organizations tend to associate the main task of managing a crisis with facilitating and allocating resources to mitigate, prepare, and respond to expected and unexpected manmade or natural hazards, the NGO sector includes all untoward events and uses the crisis term more broadly. On that note, successful organizational crisis handling depends not just on effective management, structured planning and rapid decision-making. An organization’s success relies just as much on its nature (Gilpin and Murphy, 2008) and its relationship with its employees (Frandsen and Johansen, 2011).

Since the 1960s, extensive organizational hierarchies and excessive bureaucracies have been considered ineffective (Downs, 1967). Such ways of organizing are especially true in crises. Findings show that organizations that have implemented long vertical structures often are challenged, as the vertical structure may hinder effective decision-making and often needed information flow (Berlin and Carlström, 2013). As employees can positively or negatively impact the outcome of a crisis, they should always be considered an essential resource (Frandsen and Johansen, 2011).

Everyday users of systems and procedures are often the ones that are in an immediate position to notice early warning crisis signs and detect discrepancies. Therefore, it is an essential management task to ensure that employees develop their crisis perspective and awareness to contribute to their organization’s overall crisis management (Heide and Simonsson, 2015). As argued by Weick and Ashford (2001), employees who are empowered are, in crises, not only able to act through established and rehearsed procedures; they are also capable of improvising and implementing alternative solutions. To be able to improvise and implement, there first needs to be an effort to understand connections. Such ability to turn unclear and often unexpected incidents into explicit and comprehensive situations is called sensemaking (Weick et al., 2005).

### Making Sense

In organizations, sensemaking serves as a plausible image and rationale for behavior. When stakeholders encounter ambiguous or uncertain situations, they will seek to “make sense” of them and act by examining and using existing organizational and environmental cues (Weick, 1995; Weick and Ashford, 2001; Weick et al., 2005; Maitlis and Christianson, 2014). Sensemaking is thus more about situational interpretation and action interplay than an assessment of choice (Laroche, 1995).

According to a 2014 review study by Maitlis and Christianson (2014), the “sensemaking language” was introduced in the literature at the beginning of the twentieth century in works by, e.g., Dewey (1922) and James (1890), but it was first when Karl Weick introduced the concept of organizational sensemaking in his 1969 book “The Social Psychology of Organizing” sensemaking became a critical topic within organizational research (Weick, 1969). At the time, the book contributed to an ongoing debate on whether ecological changes in an organization’s environment, among others, create modifications that engage the attention of relevant actors, resulting in recursive selections and retentions. Later, Weick (1995) described how he viewed sensemaking as a process that is “is (i) grounded in identity construction, (ii) retrospective, (iii) enactive of sensible environments, (iv) social, (v) ongoing, (vi) focused on and extracted cues and (vii) driven by plausibility rather than accuracy” (Magnussen et al., 2018, p. 247). Then, in 2001, he provided a further holistic understanding of the phenomenon by explaining the four opinion capture process stages: (1) action, (2) interaction, (3) social commitment, and (4) committed interpretation (Magnussen et al., 2018).

The notion of sensemaking may be viewed from several different perspectives. Individual sensemaking processes occur when individuals engage in retrospective and prospective thinking to construct an interpretation of reality (Sonenshein, 2010). For example, when faced with an unfolding personal crisis, a person may engage in sensemaking about their future when a situation shatters their existing personal and worldly assumptions (Keesee et al., 2008; Park, 2010). Such processes are also relevant in the study of working environments and professional interplay, examples being Weick’s known studies of the Bhopal (Weick, 2010), Mann Gulch (Weick, 1993) and Tenerife (Weick, 1990) disasters, which later have been regarded as pioneer studies within the crisis sensemaking field (Johansen et al., 2012).

The way individuals first construct meaning is influenced by several factors and levels, including their internal environment, culture, background, and identity (Prior et al., 2018). Weick (1988) argued that when people act, they initially bring constructs into existence that they set into action. In an attempt to make sense, people, through mental modeling processes, notice and bracket down the environment to identify new cues that they again contemporaneously validate (Weick et al., 2005). Cristofaro (2020) argued in his proposed *Affective-Cognitive Theory of management decisions* that when sense makers feel a positive, negative or even mixed affective state, they are driven to search for explanatory cues. However, the cues themselves are not a final solution to the sensemaking process but rather pieces of information, which the sensemaker uses to form a schema already shaped and influenced by existing “elicited affective states” (p. 9).

In later work-life studies, Christianson et al. (2009), which studied the 2003 collapse of the roof of the Baltimore & Ohio (B&O) Railroad Museum Roundhouse, found, for example, that employees’ sensemaking was triggered upon trying to understand and cope with the future of their museum and whether the collapse and destruction were to be understood as a permanent, temporary setback. In a Norwegian study on whether sensemaking processes may influence emergency call center dispatchers’ decision-making when dealing with maritime crises, Magnussen et al. (2018) found that the dispatchers` past professional experiences influenced the sensemaking processes that took place before the actual decision-making, and thus did not always result in optimal outcomes. In sum, knowledge about sensemaking enables organizations to mitigate and act when faced with a crisis (Weick et al., 2005). It provides stakeholders with a structured process of dealing with uncertainty (Weick, 1995) and explains mental reality models (Namvar et al., 2018), contributing to informed decision-making. On that note, traditional sensemaking models have been criticized for not fully considering the role of emotions in individuals and organizations (Maitlis et al., 2013).

Today, sensemaking can be viewed more as a general notion than a unified term. According to Maitlis and Christianson (2014), the many different definitions expose the many ontological assumptions (Louis, 1980; Starbuck and Milliken, 1988; Gephart, 1993; Weick, 1995) that contribute to defining and further developing sensemaking theory. However, according to the authors, it can still be argued that there are four recurring themes, which will serve as the starting point for our further discussion. First, sensemaking should be viewed as a dynamic process where the focus is on transience over constancy (Hernes and Maitlis, 2012). Second, sensemaking seems to be triggered, especially when stakeholders face unanticipated events (Maitlis, 2005). Third, despite being a general notion, sensemaking should be viewed as a social construct, as organizations and individuals make sense based on their existing thoughts and feelings, thus already being affected by the “actual, imagined, or implied presence of others “(Allport and James, 1985, p. 3, cited in Weick, 1995, p. 39 and in Maitlis and Christianson, 2014, p. 66). Finally, a fourth critical element is the fact that when people take action to make sense of a situation, it, in turn, affects the very environment they want to understand, thus creating “rational accounts of the world that enable action” (Maitlis, 2005, p. 21, cited in Maitlis and Christianson, 2014, p. 66).

## Materials and Methods

### Design

This research is a simple quantitative design, using an online survey of employees of the Norwegian Sea Rescue Society (RS).

### Population and Sample - The Case of the Norwegian Sea Rescue Society

The population for this research included the 1,600 permanent and volunteer rescue workers of The Norwegian Sea Rescue Society (Redningsselskapet, RS.) Founded in 1891, the RS is Norway’s most prominent humanitarian maritime search and rescue (SAR) organization. The Redningsselskapet organizes 52 rescue vessels, four ambulance vessels and other support vessels. In addition to national duties, the organization participates in several international projects and partnerships (Redningsselskapet, 2020). RS was deemed a relevant study sample based on its long-standing SAR traditions, organizational size, and international commitment.

Further, the organization’s response to the COVID-19 Coronavirus was considered relevant as a case study since operational insecurity, infection control requirements, and human resource challenges did pose administrative and managerial challenges to the organization. A sample size calculation was undertaken using G*Power, a free-to-use statistical software package (Faul et al., 2009). The sample size calculation was set with a statistical power of 0.80 with an alpha significance level of 0.05, and an effect size of 0.3 (Cohen, 2013). The appropriate sample size was calculated to be 82.

### Data Collection

Instead of 82 participants, 365 possible participants were sent an e-mail invitation to complete an online survey *via* the RS Human Resource department. The invitation described the study, its purpose and a hyperlink to the survey. Furthermore, contact information for the researchers, along with RS’s approval, was enclosed. One e-mail reminder was sent to potential participants, and data collection closed 16 days after the initial e-mail.

The survey was based on the Danish-developed Internal Crisis Management and Crisis Communication survey (ICMCC). This survey was designed to measure organizational participants’ perceived internal crisis management and communication levels. The ICMCC survey was developed as part of the Danish research project “Internal Crisis Management and Crisis Communication in Danish Organizations” (2011–2014), which was financed by the Danish Council for Independent Research/Social Sciences (Johansen et al., 2012). The theoretical framework of the ICMCC was built around crisis management, sensemaking and internal stakeholder theory, thus relevant to this study. The ICMCC’s homogeneity had earlier been tested by calculating Chronbach’s alpha. The test calculated an alpha value of 0.76, which was considered satisfactory according to Altman (1990).

The survey included two sections. Demographic information was collected, including the participant’s age, gender, highest educational level, years of working experience, whether they have crisis management/communication as part of their job description, and whether they had received crisis management/communication training collected. Secondly, participants were asked to pick one or more crisis definitions from a list of four predetermined from the original ICMCC survey. Then, participants were asked to rank how they perceived other employees react to crises against 18 different reaction types, using a five-point Likert scale ranging from 1 = ‘strongly disagree’ to 5 = ‘strongly agree.’

### Data Analysis

After data collection, data were cleaned and imported to Statistical Package for the Social Sciences (IBM SPSS Statistics for Windows, Version 25.0. Armonk, NY: IBM Corp). First, demographical data were analyzed using frequencies and means of central tendency for descriptive purposes. Second, the sample’s crisis perception and reactions to the crises outlined in the ICMCC survey were analyzed. Again, frequencies and central tendency were used to indicate observation averages and identify the dataset’s dispersion (Barde and Barde, 2012).

### Protection of Human Participants

To ensure that the study was performed in accordance with ethical research standards, ethical approval was obtained from the Norwegian Center for Research Data (NSD) before data collection (reference number 672295). Additionally, permission was obtained from the RS to conduct this research. Participant volunteerism was emphasized in the initial invitation to participants and during the survey. To ensure further voluntary participation, the participants answered an “I wish to participate in this study” question with a yes/no alternative as the first survey question. Additionally, to ensure anonymity, age and years of working experience, answer options were presented in predefined groupings.

## Findings

### Demographics

A total of 73 (*N* = 73) persons agreed to participate in this study. The response rate was 20%. That gave an overall statistical power of 0.75. Seventy-seven percent of the respondents were males, and 23% were females. Most participants belonged to the 50–59 age group (33.5%) or the 40–49 (33.0%). Further, 19% belonged to the 30–39 group, 5.5% to the 20–29, and 11.0% to the 60–69 age group. Over half (57.8%) stated four years of higher education as their highest level, while 18.3% listed a high-school level. Twenty-two percent had more than four years of higher education, while one individual listed a doctoral educational level. Years of RS working experience varied from under one to 15+. The distributions were as followed: 0–1 (10.7%), 1–5 (25.3%), 6–10 (36.0%), 11–15 (13.3%), and 15+ (14.7%). Close to half (48.5%) either strongly or somewhat agreed that they had crisis management as part of their function. Thirty-four point 5% either strongly or somewhat disagreed. Over half (55.4%) strongly disagreed that they had received crisis management/communication training, while 9.5% somewhat disagreed. Thirty-one point 1% either strongly or somewhat agreed (Table 1).

**Table 1:** Demographics.

Gender	%	Age group	%	Years of RS experience	%	Educational level	%
Male	77	20–29	5.5	0–1	10.7	High-school	18.3
Female	23	30–39	19.0	1–5	23.3	Higher (4 years)	57.8
		40–49	33.0	6–10	36.0	Higher (4+ years)	22.5
		50–59	33.5	11–15	13.3	Doctoral level	1.4
		60–69	11.0	15+	14.7		

*N = 73.*

### Employee’s Pattern Perception of Crisis

Of the four crisis pattern descriptions provided (Table 2), over half (63.6%) supported the claim that a crisis is an incident involving damage to stakeholders (customers, members, employees, volunteers, etc.). Fifty-four point 5% supported the assertion that an incident put parts of the organization out of operation within a short period. Fewer of the sample population perceived that a crisis threatens the entire organization’s existing foundation (22.1%) or is poorly handled by the organization’s management (10.4%).

**Table 2:** Employee’s pattern perception of crisis (from high to low).

Crisis perception	%
An incident involving damage to stakeholders (customers, members, employees, volunteers, etc.)	63.6
An incident that within a short time-period put parts of the organization out of operation	54.5
An incident that threatens the entire organization’s existing foundation	22.1
An incident that is poorly handled by the management of the organization	10.4

*N = 73.*

### Perceived Co-worker’s Reaction to Crises

Upon being asked how the participants perceived that their co-workers react to crises (Table 3), most perceived they would out a need for more information (*M* = 4 0.32, SD = 0.92). Next followed a sense that several would produce more informal communication (*M* = 2.90, SD = 1.16), feel insecure (*M* = 2.89, SD = 1.20), and frustrated (*M* = 2.86, SD = 1.23). On the other side, as seen in the table, few perceived that their co-workers would become passive (*M* = 1.70, SD = 0.99) or panic (*M* = 1.44. SD = 0.77). Further, the findings showed that only a minory perceived that their colleagues would lose motivation (*M* = 1.83, SD = 1.08), leave the organization (*M* = 1.80, SD = 1.01), lose confidence (*M* = 1.79, SD = 1.06), or feel ashamed (*M* = 1.73, SD = 0.95).

**Table 3:** Perceived reactions to crises.

Text	Mean	SD
Need more information	4.32	0.92
More informal communication	2.90	1.16
Insecurity	2.89	1.20
Frustration	2.86	1.23
Spread rumors	2.75	1.28
Feel sorrow	2.63	0.99
Misunderstand the situation	2.48	1.08
Scared	2.29	1.33
Become silent	2.25	1.04
Community	2.13	1.29
Loss of motivation	1.83	1.08
Leaving the organization	1.80	1.01
Loss of confidence	1.79	1.06
Feel betrayed	1.77	0.95
Feel ashamed	1.73	0.95
Passive	1.70	0.99
Identification	1.69	0.89
Panic	1.44	0.77

## Discussion

First, by taking as a starting point that sensemaking involves a dynamic process where the focus is on transience over constancy (Hernes and Maitlis, 2012), it flows nicely together with the notion that a crisis, here the COVID-19 pandemic, in its nature, is a rare, overwhelming and abnormal occurrence. Most participants perceived the pandemic as an incident involving damage to stakeholders (customers, members, employees, volunteers, etc.). That over half also defined the ongoing corona crisis as an incident that within a short time put parts of the organization out of operation; it also aligned well with Pearson and Clair's (1998) definition of an organizational crisis, thus being a low-probability, high-impact event. Based on the assumption that the sample responded to the survey questions built on how they perceived the Norwegian Sea Rescue Society had responded to the COVID-19 outbreak during the first three quarters of 2020, only 10.4% reported that they associated a crisis with an incident that was poorly handled by the management. Despite that managing crisis can be quite challenging and stressful compared to standard routine procedures, the findings indicate strong confidence in RS’s ability to handle a crisis. Combined, it indicates trust in management and widespread belief in organizational resilience, that RS is an organization that internally can handle both abrupt and cumulative incidents (Hwang and Lichtenthal, 2000), which both are represented in the ongoing pandemic. Such findings are positive in light of Gilpin and Murphy's (2008) earlier discussed argument of how an organization’s success relies on its nature and its relationship to its workers (Frandsen and Johansen, 2011). Findings signal a highly empowered employee group with a high degree of crisis awareness (Weick and Ashford, 2001), who can turn unclear and often unexpected incidents into understandable and tangible situations (Weick et al., 2005).

Second, results align with the assumption that sensemaking seems to be triggered, especially when stakeholders face unanticipated events (Maitlis, 2005). That the top three found perceived reaction patterns in this study were to (1) seek out more information, (2) engage in more informal communication, and (3) experience a feeling of insecurity supports the assumptions of, among others (Weick, 1995; Weick et al., 2005) and Maitlis and Christianson (2014), which argued that when stakeholders encounter ambiguous or uncertain situations, they will seek to “make sense” of them through the use of existing organizational knowledge, networks and experiences. However, the search for explanatory cues is not always the final solution to the sensemaking process but rather pieces of information, which the sensemaker uses to form a schema that is often already shaped and influenced by existing states. Therefore, as argued by Cristofaro (2020), it is necessary to focus more on the role of affective states in determining possible cognition errors. That said, making sense of a crisis is not always easy as such an incident presents stakeholders with higher uncertainty levels (Mitroff et al., 1987) and limited information flow, often based upon less validated materials (Lu and Xue, 2016). Therefore, traditional information networks may not always prove sufficient, resulting in that co-workers seeking out information elsewhere.

While informal communication networks may have several benefits, there is an imminent danger that employees may fall victim to an illicit or little nuanced information flow. Combined with a higher degree of uncertainty, such information may negatively affect employees’ sensemaking processes about their current and future (Keesee et al., 2008; Park, 2010). Signs of such negative ongoing processes are also identifiable in this study, as co-workers are, by their peers, perceived to show somewhat signs of misunderstanding, frustration, and insecurity, and some are also, to a degree, perceived to feel sorrow and fear. On a positive note, few perceived that their co-workers encountered a loss of motivation or wanted to leave the organization in a crisis. This may suggest that the Norwegian Sea Rescue Society (RS) is a resilient organization with the ability to counteract and adjust to triggering events. Emotional response findings also support the notion of organizational robustness. Few perceived that their co-workers reacted with a feeling of shame or betrayal when their organization experienced a crisis. On the contrary, the results indicate that RS employees consider their co-workers to handle crises well, as few perceived that typical crisis reaction patterns involved high degrees of passivity, silence, panic or loss of confidence.

Third, despite being a general notion, sensemaking can be viewed as a social construct, as organizations and individuals make sense based on their existing thoughts and feelings, thus already being affected by the “actual, imagined, or implied presence of others” (Allport and James, 1985, p. 3, cited in Weick, 1995, p. 39 and in Maitlis and Christianson, 2014, p. 66). As seen here, employees can positively or negatively impact the outcome of a crisis (Frandsen and Johansen, 2011) through their actions and behavior. Therefore, as Heide and Simonsson (2015) discussed, it is an essential management task to ensure that employees develop relevant crisis understanding, perspective, and awareness. An interesting observation in this study was that close to half of the sample, or more specifically, 35 individuals, while not belonging to the top management, perceived that they had crisis management and communication as part of their formal function. On the one hand, such high numbers may indicate an organization with unclear internally communicated roles and responsibilities. Conversely, the finding may reflect a relatively flat and transparent organizational structure, where top management trusts their employees and the employees take active ownership and contribute to their organization’s overall crisis management.

Finally, a fourth critical element is that when people take action to make sense of a situation, it, in turn, affects the very environment they want to understand, thus creating “rational accounts of the world that enable action” (Maitlis, 2005, p. 21, cited in Maitlis and Christianson, 2014, p. 66). That well over half of the sample population had been with the organization for 6 years indicates That the Norwegian Sea Rescue Society (RS) has a stable employee pool. While a stable pool can be a strength both in everyday operations and in crisis, it can also result in extensive hierarchies, ingrained cultures and traditions that get in the way of effective crisis response (Downs, 1967; Berlin and Carlström, 2013). As employees often are the ones that are in the immediate position to notice early warning crisis signs and detect discrepancies, it is an essential management task to ensure that the employees develop and keeps up to date on their crisis sensemaking and awareness skills. As argued by Weick and Ashford (2001), employees who are empowered are, in crises, not only able to act through established and rehearsed procedures; they are also capable of improvising and implementing alternative solutions.

## Conclusion, Recommendations and Limitations

Findings showed that a majority perceived a crisis as an incident involving damage to stakeholders and that it was an incident that put parts of the organization out of operation within a short period. Fewer perceived that a crisis threatens the entire organization’s existing foundation or is poorly handled by the organization’s management. The results indicated that most of the sampled population strongly believed in their organization’s overall resilience level, thus its ability to react and recover from damaging events or blows. However, the results also indicated a somewhat vague understanding of internal roles and responsibilities. Their need for more information became evident in co-workers’ reaction patterns. The sample perceived that their co-workers engaged in informal communication and rumor spreading. Signs of ongoing negative processes were also identifiable in this study, as co-workers were by their peers perceived to show somewhat signs of misunderstanding, frustration, and insecurity. Crisis perception, knowledge of own organization, limitations and capabilities, roles, and responsibilities are important factors in crisis management that should be enhanced through communication and information sharing to prevent spreading rumors and functional disruption in an organization during a crisis. This paper deals with Norwegian employees. However, these findings’ implications are global and include necessary educational initiatives and research focusing on employees’ perceptions of- and reactions to an organizational crisis. More research, preferably with the same approach, is recommended to gain further knowledge on how employees perceive and react to organizational crises. We recommend that future studies examine the relationships between variables using renowned statistical methods.

This study has several limitations. First, this study was limited in scope as data was collected from a limited sample population and a relatively short period. However, the response rate with associated achieved statistical power of 0.75 is close to the desired target of 0.80, hence giving a good indication of the current perceived understanding of crises and their responses. Second, the sample was presented only with predefined options, thus not providing individual options to define the different terms. Third, the sample had to interpret terms like crises, panic, and insecurity individually, which may cause lower term validity. Fourth, since the study was done during an ongoing pandemic, there is a bias in terms of the amount of information that existed at the time data was collected. Fifth, as this study focuses on individual perceptions only, there will always be a risk of bias or other barriers to perceptual accuracy. Finally, it should be noted that the original study was conducted on the organizational level among the 367 largest private companies and 98 public municipalities in Denmark (Johansen et al., 2012), while in this study, the same survey was used on a single organization and applied on the individual level. We still deem using the same instrument relevant, as it measures the participant’s individual perceptions in both studies.

## Data Availability Statement

The raw data supporting the conclusions of this article will be made available by the authors, without undue reservation. The data is in Norwegian language only.

## Ethics Statement

The studies involving human participants were reviewed and approved by NSD - Norwegian Centre for Research Data. The patients/participants provided their written informed consent to participate in this study.

## Author Contributions

JS: conceptualization, methodology, formal analysis, visualization, and project administration. JS, JR, LG, AK-M, KG, and AH: writing—original draft preparation and writing—review and editing. All authors contributed to the article and approved the submitted version.

## Conflict of Interest

The authors declare that the research was conducted in the absence of any commercial or financial relationships that could be construed as a potential conflict of interest.

## Publisher’s Note

All claims expressed in this article are solely those of the authors and do not necessarily represent those of their affiliated organizations, or those of the publisher, the editors and the reviewers. Any product that may be evaluated in this article, or claim that may be made by its manufacturer, is not guaranteed or endorsed by the publisher.

## References

[ref1] Adamu A. A. Mohamad B. (2019). Developing a strategic model of internal crisis communication: empirical evidence from Nigeria. Int. J. Strateg. Commun. 13, 233–254. doi: 10.1080/1553118X.2019.1629935

[ref2] Aljuhmani H. Y. Emeagwali O. L. (2017). The roles of strategic planning in organizational crisis management: The case of Jordanian banking sector. Int. Rev. Manag. Mark. 7, 50–60.

[ref001] Allport G. James W. (1985). Psychology: The Briefer Course. University of Notre Dame Press.

[ref3] Altman D. G. (1990). Practical Statistics for Medical Research. Florida: CRC press.

[ref4] Ansell C. Boin A. Keller A. (2010). Managing transboundary crises: identifying the building blocks of an effective response system. J. Conting. Crisis Manag. 18, 195–207. doi: 10.1111/j.1468-5973.2010.00620.x

[ref5] Bailey K. Breslin D. (2021). The COVID-19 pandemic: what can we learn from past research in organizations and management? Int. J. Manag. Rev. 23, 3–6. doi: 10.1111/ijmr.12237

[ref6] Barde M. P. Barde P. J. (2012). What to use to express the variability of data: standard deviation or standard error of mean? Perspect. Clin. Res. 3, 113–116. doi: 10.4103/2229-3485.100662, PMID: 23125963 PMC3487226

[ref002] Barton A. H. (1970). Communities in Disaster: A Sociological Analysis of Collective Stress Situations. Ward Lock Educational.

[ref8] Berlin J. M. Carlström E. D. (2013). The dominance of mechanistic behaviour: a critical study of emergency exercises. Int. J. Emerg. Manag. 9, 327–350. doi: 10.1504/IJEM.2013.059878

[ref9] Boin A. t Hart P. (2022). From Crisis to reform? Exploring three post-COVID pathways. Pol. Soc. 41, 13–24. doi: 10.1093/polsoc/puab007

[ref10] Bundy J. Pfarrer M. D. Short C. E. Coombs W. T. (2017). Crises and crisis management: integration, interpretation, and research development. J. Manag. 43, 1661–1692. doi: 10.1177/0149206316680030

[ref11] Burns T. Stalker G.M. (1961). The Management of Innovation. London: Tavistock.

[ref12] Christianson M. K. Farkas M. T. Sutcliffe K. M. Weick K. E. (2009). Learning through rare events: significant interruptions at the Baltimore & Ohio Railroad Museum. Organ. Sci. 20, 846–860. doi: 10.1287/orsc.1080.0389

[ref13] Cohen J. (2013). Statistical Power Analysis for the Behavioral Sciences. London: Academic press.

[ref14] Coombs W. T. Holladay S. J. (2014). How publics react to crisis communication efforts: comparing crisis response reactions across sub-arenas. J. Commun. Manag. 18, 40–57. doi: 10.1108/JCOM-03-2013-0015

[ref15] Cristofaro M. (2020). "I feel and think, therefore I am": An affect-cognitive theory of management decisions. Eur. Manag. J. 38, 344–355. doi: 10.1016/j.emj.2019.09.003

[ref17] Dewey J. (1922). Human Nature and Conduct. Mineola, NY: Dover.

[ref18] Downs A. (1967). Inside Bureaucracy. New York: Little Brown.

[ref003] Fagerland O. M. B. (2020). Internal Crisis Communication: Case of The Norwegian Sea Rescue Society (Master’s thesis, University of South-Eastern Norway).

[ref19] Faul F. Erdfelder E. Buchner A. Lang A.-G. (2009). Statistical power analyses using G* power 3.1: tests for correlation and regression analyses. Behav. Res. Methods 41, 1149–1160. doi: 10.3758/BRM.41.4.1149, PMID: 19897823

[ref20] Frandsen F. Johansen W. (2011). The Study of Internal Crisis Communication: Towards an Integrative Framework. Corporate Communications: An International Journal.

[ref21] Gephart R. P. (1993). The textual approach: risk and blame in disaster sensemaking. Acad. Manag. J. 36, 1465–1514. doi: 10.5465/256819

[ref22] Gilpin D. R. Murphy P. J. (2008). Crisis Management in a Complex World. London: Oxford University Press.

[ref23] Heide M. Simonsson C. (2015). Struggling with internal crisis communication: a balancing act between paradoxical tensions. Pub. Relat. Inq. 4, 223–255. doi: 10.1177/2046147X15570108

[ref24] Hernes T. Maitlis S. (2012). Process, Sensemaking, and Organizing. London: Oxford University Press.

[ref25] Hwang P. Lichtenthal J. D. (2000). Anatomy of organizational crises. J. Conting. Crisis Manag. 8, 129–140. doi: 10.1111/1468-5973.00132

[ref26] James W. (1890). The Principles of Psychology. New York: Dover.

[ref27] Johansen W. Aggerholm H. K. Frandsen F. (2012). Entering new territory: a study of internal crisis management and crisis communication in organizations. Public Relat. Rev. 38, 270–279. doi: 10.1016/j.pubrev.2011.11.008

[ref28] Johansen W. Frandsen F. (2007). Crisis Communication: When the Corporate Image and Reputation Is Threatened [English Trans.]. Frederiksberg, Denmark: Samfundslitteratur.

[ref29] Keesee N. J. Currier J. M. Neimeyer R. A. (2008). Predictors of grief following the death of one's child: The contribution of finding meaning. J. Clin. Psychol. 64, 1145–1163. doi: 10.1002/jclp.20502, PMID: 18698614

[ref30] Kerchner C. T. Schuster J. H. (1982). The uses of crisis: taking the tide at the flood. Rev. High. Educ. 5, 121–141. doi: 10.1353/rhe.1982.0011

[ref31] Khorram-Manesh A. Carlström E. Hertelendy A. Goniewicz K. Casady C. Burkle F. (2020). Does the prosperity of a country play a role in COVID-19 outcomes? Disaster Med. Public Health Prep. 16, 177–186. doi: 10.1017/dmp.2020.304, PMID: 32782059 PMC7477401

[ref32] Kim Y. (2018). Enhancing employee communication behaviors for sensemaking and sensegiving in crisis situations: strategic management approach for effective internal crisis communication. J. Commun. Manag. 22, 451–475. doi: 10.1108/JCOM-03-2018-0025

[ref33] Klein J. (2021). “Infectious disease, pandemics, and education: From miasms to COVID-19,” in Meeting the Challenges of Existential Threats Through Educational Innovation. ed. H. Saeverot (Routledge), 85–97.

[ref34] Kovoor-Misra S. Clair J. A. Bettenhausen K. L. (2001). Clarifying the attributes of organizational crises. Technol. Forecast. Soc. Chang. 67, 77–91. doi: 10.1016/S0040-1625(99)00081-5

[ref35] Lalonde C. Roux-Dufort C. (2013). Challenges in teaching crisis management: connecting theories, skills, and reflexivity. J. Manag. Educ. 37, 21–50. doi: 10.1177/1052562912456144

[ref36] Laroche H. (1995). From decision to action in organizations: decision-making as a social representation. Organ. Sci. 6, 62–75. doi: 10.1287/orsc.6.1.62

[ref37] Linnenluecke M. K. (2017). Resilience in business and management research: A review of influential publications and a research agenda. Int. J. Manag. Rev. 19, 4–30. doi: 10.1111/ijmr.12076

[ref38] Longstaff P. H. (2005). Security, Resilience, and Communication in Unpredictable Environments Such as Terrorism, Natural Disasters, and Complex Technology. England: Center for Information Policy Research, Harvard University.

[ref39] Louis M. R. (1980). Surprise and sensemaking: what newcomers experience in entering unfamiliar settings. Adm. Sci. Q. 25, 226–251. doi: 10.2307/2392453, PMID: 10247029

[ref40] Lu X. Xue L. (2016). Managing the unexpected: sense-making in the Chinese emergency management system. Public Adm. 94, 414–429. doi: 10.1111/padm.12261

[ref41] Magnussen L. I. Carlstrøm E. Berge A.-K. Wegger F. Sørensen J. L. (2018). Help we are sinking! Stories from Norwegian dispatch centers on decision-making in unfamiliar and ambiguous situations. J. Emerg. manag. 16, 245–254. doi: 10.5055/jem.2018.0373 30234910

[ref42] Maitlis S. (2005). The social processes of organizational sensemaking. Acad. Manag. J. 48, 21–49. doi: 10.5465/amj.2005.15993111

[ref43] Maitlis S. Christianson M. (2014). Sensemaking in organizations: taking stock and moving forward. Acad. Manag. Ann. 8, 57–125. doi: 10.5465/19416520.2014.873177

[ref44] Maitlis S. Vogus T. J. Lawrence T. B. (2013). Sensemaking and emotion in organizations. Organ. Psychol. Rev. 3, 222–247. doi: 10.1177/2041386613489062

[ref45] Milburn T. W. Schuler R. S. Watman K. H. (1983). Organizational crisis. Part I: definition and conceptualization. Hum. Relat. 36, 1141–1160. doi: 10.1177/001872678303601205

[ref46] Mitroff I. I. Shrivastava P. Udwadia F. E. (1987). Effective crisis management. Acad. Manag. Perspect. 1, 283–292. doi: 10.5465/ame.1987.4275639

[ref47] Namvar M. Cybulski J. Phang C. Wee C.-Y. Tan K. Y. (2018). Simplifying sensemaking: concept, process, strengths, shortcomings, and ways forward for information systems in contemporary business environments. Australas. J. Inf. Syst. 22:1645. doi: 10.3127/ajis.v22i0.1654

[ref48] Norwegian Government (2020). Omfattende tiltak for å bekjempe koronaviruset. Available at: https://www.regjeringen.no/no/aktuelt/nye-tiltak/id2693327/ (Accessed November 11, 2020).

[ref49] Norwegian Institute of Public Health (2020). Status koronavirus 12. mars 2020. Available at: https://www.fhi.no/nyheter/2020/status-koronavirus-12.-mars-2020/ (Accessed November 11, 2020).

[ref50] Nystrom P. Starbuck W. H. (2015). To Avoid Organizational Crises, Unlearn. Available at: https://ssrn.com/abstract=2708289

[ref51] Park C. L. (2010). Making sense of the meaning literature: an integrative review of meaning making and its effects on adjustment to stressful life events. Psychol. Bull. 136, 257–301. doi: 10.1037/a0018301, PMID: 20192563

[ref52] Pearson C. M. Clair J. A. (1998). Reframing crisis management. Acad. Manag. Rev. 23, 59–76. doi: 10.2307/259099

[ref53] Pearson C. M. Mitroff I. I. (1993). From crisis prone to crisis prepared: a framework for crisis management. Acad. Manag. Perspect. 7, 48–59. doi: 10.5465/ame.1993.9409142058

[ref54] Peyravi M. Marzaleh M. A. Khorram-Manesh A. (2021). “A disaster: Genral principles and management,” in Handbook of Disaster and Emergency Management. 2nd Edn. eds. Khorram-Manesh A. Goniewicz K. Hertelendy A. Dulebenets M. (Göteborg: Kompendiet).

[ref55] Prior D. D. Keränen J. Koskela S. (2018). Sensemaking, sensegiving and absorptive capacity in complex procurements. J. Bus. Res. 88, 79–90. doi: 10.1016/j.jbusres.2018.03.009

[ref56] Redningsselskapet (2020). Welcome to the Norwegian sea rescue society. Available at: https://www.redningsselskapet.no/english/ (Accessed November 11, 2020).

[ref57] Roux-Dufort C. Lalonde C. (2013). Exploring the Theoretical Foundations of Crisis Management. J. Conting. Crisis Manag. 21, 1–3.

[ref58] Seidl D. Werle F. (2018). Inter-organizational sensemaking in the face of strategic meta-problems: requisite variety and dynamics of participation. Strateg. Manag. J. 39, 830–858. doi: 10.1002/smj.2723

[ref59] Smart C. Vertinsky I. (1984). Strategy and the environment: A study of corporate responses to crises. Strateg. Manag. J. 5, 199–213. doi: 10.1002/smj.4250050302

[ref60] Sonenshein S. (2010). We're changing—Or are we? Untangling the role of progressive, regressive, and stability narratives during strategic change implementation. Acad. Manag. J. 53, 477–512. doi: 10.5465/amj.2010.51467638

[ref61] Sørensen J. L. (2017). Norwegian maritime crisis collaboration exercises: are they useful? Doctoral dissertation, Northcentral University.

[ref62] Sriharan A. Hertelendy A. J. Banaszak-Holl J. Fleig-Palmer M. M. Mitchell C. Nigam A.. (2022). Public health and health sector crisis leadership during pandemics: a review of the medical and business literature. Med. Care Res. Rev. 79, 475–486. doi: 10.1177/10775587211039201 34474606 PMC9218413

[ref63] Starbuck W. H. Milliken F. J. (1988). “Executives' perceptual filters: what they notice and how they make sense,” in The Executive Effect: Concepts and Methods for Studying Top Managers. ed. Hambrick D. C. (Greenwich, CT: JAI Press), 35–65.

[ref64] Steen R. Morsut C. (2020). Resilience in crisis management at the municipal level: The synne storm in Norway. Risk, Hazards Crisis Pub. Pol. 11, 35–60. doi: 10.1002/rhc3.12178

[ref65] Tan B. Pan S. L. Chen W. Huang L. (2020). Organizational Sensemaking in ERP implementation: The influence of Sensemaking structure. MIS Q. 44, 1773–1809. doi: 10.25300/MISQ/2020/11872

[ref66] Teo W. L. Lee M. Lim W. S. (2017). The relational activation of resilience model: how leadership activates resilience in an organizational crisis. J. Conting. Crisis Manag. 25, 136–147. doi: 10.1111/1468-5973.12179 PMC7166971 40476945

[ref67] Van Wart M. Kapucu N. (2011). Crisis management competencies: The case of emergency managers in the USA. Public Manag. Rev. 13, 489–511. doi: 10.1080/14719037.2010.525034

[ref68] Weick K. E. (1969). The Social Psychology of Organizing. Reading, MA: Addison-Wesley.

[ref69] Weick K. E. (1988). Enacted sensemaking in crisis situations [1]. J. Manag. Stud. 25, 305–317. doi: 10.1111/j.1467-6486.1988.tb00039.x

[ref70] Weick K. E. (1990). The vulnerable system: an analysis of the Tenerife air disaster. J. Manag. 16, 571–593. doi: 10.1177/014920639001600304

[ref71] Weick K. E. (1993). The collapse of sensemaking in organizations: The Mann gulch disaster. Adm. Sci. Q. 38, 628–652. doi: 10.2307/2393339

[ref72] Weick K. E. (1995). Sensemaking in Organizations. United States: Sage.

[ref73] Weick K. E. (2010). Reflections on enacted sensemaking in the Bhopal disaster. J. Manag. Stud. 47, 537–550. doi: 10.1111/j.1467-6486.2010.00900.x

[ref74] Weick K. E. (2012). Making Sense of the Organization, Volume 2: The Impermanent Organization. New Jersey: John Wiley & Sons.

[ref75] Weick K. E. (2015). The social psychology of organizing. Management 18:189.

[ref76] Weick K. E. Ashford S. J. (2001). Learning in organizations. New Handbook of Organ. Commun. Adv. Theor. Res. Methods 704:731.

[ref77] Weick K. E. Sutcliffe K. Obstfeld D. (2005). Organizing and the process of sensemaking. Organ. Sci. 16, 409–421. doi: 10.1287/orsc.1050.0133

[ref78] Williams T. A. Gruber D. A. Sutcliffe K. M. Shepherd D. A. Zhao E. Y. (2017). Organizational response to adversity: fusing crisis management and resilience research streams. Acad. Manag. Ann. 11, 733–769. doi: 10.5465/annals.2015.0134

[ref79] Winston B. E. (ed.) (2019). “Leadership growth through crisis: an investigation of leader development during tumultuous circumstances,” in Communicating Through Crisis. Switzerland: Palgrave.

[ref80] Wolbers J. Kuipers S. Boin A. (2021). A systematic review of 20 years of crisis and disaster research: trends and progress. Risk, Hazards Crisis Pub. Pol. 12, 374–392. doi: 10.1002/rhc3.12244

[ref81] Zamoum K. Gorpe T. S. (2018). “Crisis management: a historical and conceptual approach for a better understanding of today's crises,” in Crisis Management-Theory and Practice. eds. Holla, K. Titko M. Ristvej J. (United Kingdom: IntechOpen).

